# Effect of localized helium ion irradiation on the performance of synthetic monolayer MoS_2_ field-effect transistors

**DOI:** 10.3762/bjnano.11.117

**Published:** 2020-09-04

**Authors:** Jakub Jadwiszczak, Pierce Maguire, Conor P Cullen, Georg S Duesberg, Hongzhou Zhang

**Affiliations:** 1 School of Physics, Trinity College Dublin, Dublin 2, Ireland; 2 School of Chemistry, Trinity College Dublin, Dublin 2, Ireland; 3 State Institute of Physics, EIT 2, Faculty of Electrical Engineering and Information Technology, Universität der Bundeswehr München, Werner-Heisenberg-Weg 39, 85577 Neubiberg, Germany

**Keywords:** 2D materials, contacts, defect engineering, helium ion microscope, ion beam doping, vacancies, two-dimensional materials

## Abstract

Helium ion irradiation is a known method of tuning the electrical conductivity and charge carrier mobility of novel two-dimensional semiconductors. Here, we report a systematic study of the electrical performance of chemically synthesized monolayer molybdenum disulfide (MoS_2_) field-effect transistors irradiated with a focused helium ion beam as a function of increasing areal irradiation coverage. We determine an optimal coverage range of approx. 10%, which allows for the improvement of both the carrier mobility in the transistor channel and the electrical conductance of the MoS_2_, due to doping with ion beam-created sulfur vacancies. Larger areal irradiations introduce a higher concentration of scattering centers, hampering the electrical performance of the device. In addition, we find that irradiating the electrode–channel interface has a deleterious impact on charge transport when contrasted with irradiations confined only to the transistor channel.

## Introduction

Layered two-dimensional (2D) semiconductors have come to the fore in recent years as promising candidates for the implementation of flexible, transparent, and low-power electronics. In particular, transition metal dichalcogenides (TMDs), such as molybdenum disulfide (MoS_2_), have demonstrated impressive on/off ratios (approx. 10^7^) in field-effect transistors (FETs), while maintaining carrier mobilities that may be adequate for commercial applications [1–2]. At the same time, advances in chemical vapor deposition (CVD) techniques have allowed for the reliable millimeter-scale synthesis of well-performing monolayer TMD films [3–5], leading to viable large-scale integration of on-chip TMD FETs. With device miniaturization, it becomes key to understand the impact of defects such as chalcogen vacancies on the electrical transport properties of FETs based on 2D semiconductors. This is particularly crucial for device applications in radiation-rich environments (e.g., space satellite technologies), since defects can be introduced by ionizing particle irradiation while the devices are in continuous operation.

Recently, noble gas ion beam irradiation has opened the field to the exploration of nanometer-scale structural modifications of TMD devices [6–8]. The localized formation of defects by focused ion beam irradiation has been shown to induce unusual electronic properties in monolayer TMDs, such as pseudo-metallic phase transitions in MoS_2_ and WSe_2_ [9–10], resistive switching in MoS_2_ [11], as well as enhanced out-of-plane charge transfer in 2D graphene/WSe_2_ heterostructures [12]. Energetic light ions are known to preferentially sputter chalcogen atoms from TMDs while retaining an adequate micrometer-scale structural integrity for irradiation doses up to approx. 10^16^ ions cm^−2^ [13–17], as well as good electrical conductivity for up to approx. 10^18^ ions cm^−2^ [9–10,18]. Sulfur vacancies (SVs) and the formation of a dislocation–divacancy complex can lead to significant n-doping in MoS_2_ [19], which shifts the threshold voltage (*V*_th_) of the FET to higher negative gate biases [20–21]. These complex states may also improve the carrier mobility across a given gate bias range by forming stable impurity bands near the conduction band [22]. Some theoretical studies suggest, however, that individual SVs ought to act as electron acceptors in MoS_2_ [23–24]. As the spread of a typical focused He^+^ ion probe is several nanometers, the formation of other defects in the irradiated 2D crystal lattice is also expected [25–26], which may bring about the often-observed negative shifts of the MoS_2_ FET threshold voltage after ion irradiation. Such n-type doping behavior achieved by selective ion sputtering in thin film transistors has also been observed in He^+^-irradiated InGaZnO devices [27]. This irradiation-induced carrier activation depends not only on the fluence of the ion beam, but also on the absolute number of defects that can be introduced. Therefore, the creation of defects in TMD FETs may serve to improve charge transport and tune the device performance [10,20,28], while the effective donor concentration and the introduced scattering potentials need to be tailored accordingly [29–30]. Thus far, these studies have focused either on spatially confined defect generation, or full-channel modifications in TMD FETs. A research space exists for exploring the intermediate regime between these two extrema by finely controlling the area over which defects are seeded. For practical devices, it is also important to consider the effect of particle irradiation on the deposited metal–semiconductor contact interface. Recent work has shown that irradiation-induced heating of the electrode area can reverse majority carrier polarity in MoTe_2_ [31], while pre-treatment with a broad-beam argon ion source can decrease the contact resistance of Ni-MoS_2_ two-fold [32]. In this paper, we investigate the effect of the defect population on the performance of MoS_2_ FETs via varying the area of ion irradiation in the FET channel. We also examine the performance of devices upon irradiation of one of the electrical contact interfaces.

## Experimental

**Figure 1:**
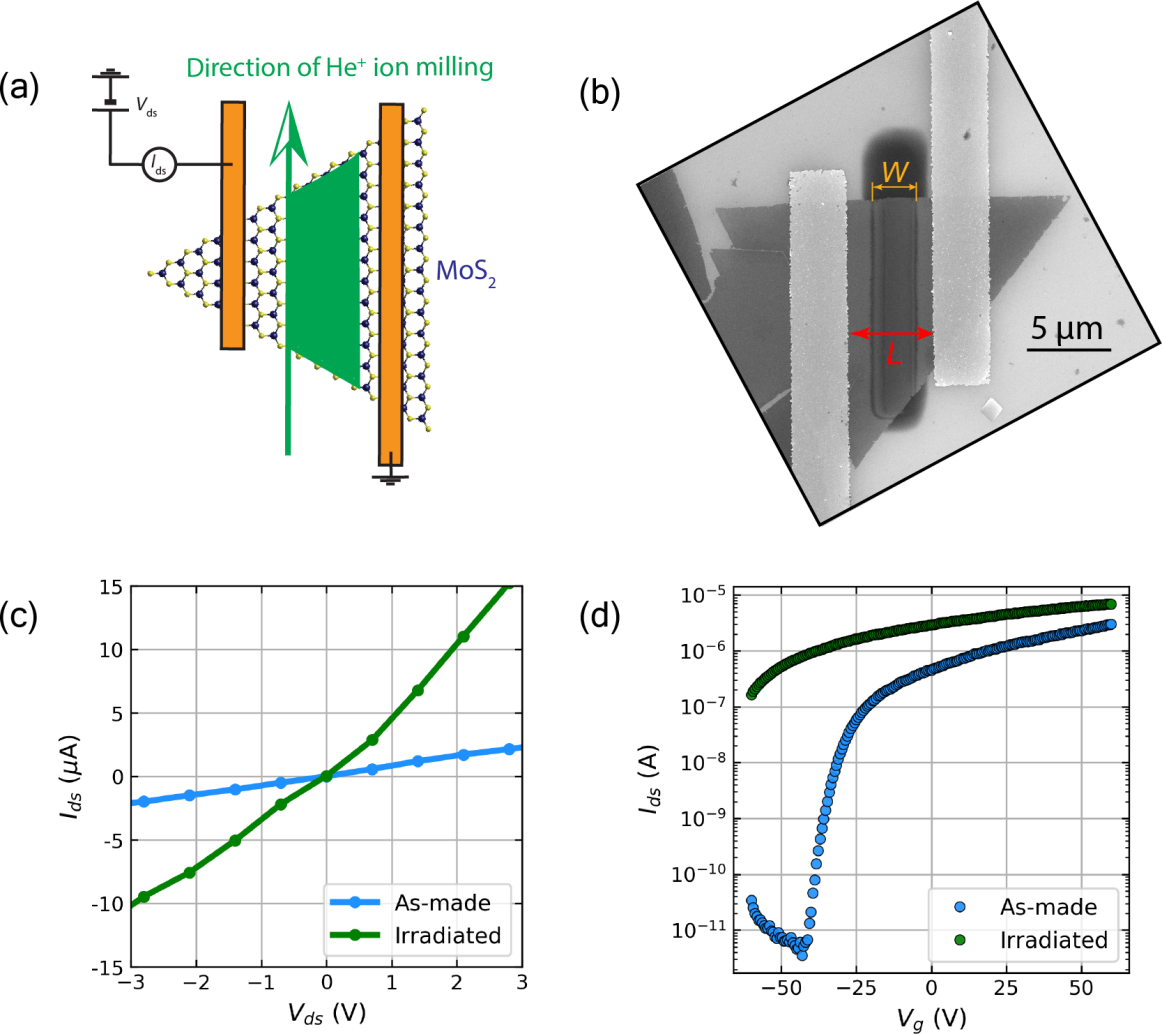
Experimental design and basic electrical characteristics. (a) Sketch demonstrating the irradiation strategy on contacted CVD-grown monolayer MoS_2_ devices. The green area marks the designed irradiation area. (b) SEM image of an irradiated device. *W* marks the width of the exposed region, while *L* is the length of the FET channel. *L* is 5 μm in the image. (c) *I*–*V* curve of a device after irradiation (corresponding to *I*_R_ = 7%). (d) Transfer curve of the same device demonstrating reduced gate tunability and a higher electrical conductance after He^+^ ion irradiation.

Monolayer MoS_2_ samples were synthesized using a CVD microreactor method, described in detail in [33], directly on 285 nm SiO_2_/Si substrates, which also served as the back-gate in the FET configuration. MoS_2_ flakes were contacted with electrodes using standard electron beam lithography on polymethyl methacrylate (PMMA) resist, followed by lift-off in acetone and metal deposition by evaporation (5 nm Ti and 35 nm Au). Electrical testing was carried out using a dual-channel source–measure unit connected to tungsten micromanipulator probes (Imina miBot) in the vacuum chamber of a customized Zeiss EVO scanning electron microscope (SEM). Prior to testing, the devices were outgassed at a pressure of approx. 10^−5^ mbar for 12 h to minimize surface adsorbates. Helium ion irradiations were carried out at a beam energy of 30 keV and He gas pressure of 2 × 10^−6^ Torr, using a Zeiss Nanofab microscope. The fabricated MoS_2_ FETs were placed in the helium ion microscope chamber (after initial electrical testing to confirm functionality) and were irradiated with the stage tilt angle set to 0°. At this angle of incidence, the helium ion beam ought to produce sulfur vacancies chiefly in the bottom sulfuric layer of the SiO_2_-supported MoS_2_ flake [34]. The average recorded beam current throughout the irradiations was 37.5 ± 0.4 pA, and the probe size was determined at approx. 7 nm [9]. The areal ion dose delivered to each sample was maintained at approx. 10^17^ ions cm^−2^, with a step size of 1 nm and a dwell time of 4.3 μs throughout the duration of a unidirectional probe scan. Post-irradiation SEM imaging was carried out in a Zeiss Supra microscope using the in-lens detector at a low beam energy of less than 5 keV to ensure a strong surface sensitivity. Figure 1a shows a sketch of the experimental geometry. A SEM micrograph of a typical irradiated device is also presented in Figure 1b. The marked distances *W* and *L* denote the width of the irradiated region and the length of the FET channel, respectively. *L* was kept constant at 5 μm in this work, while *W* was varied to obtain a designated irradiation-to-channel ratio, *I*_R_ = *W*/*L*. Following the irradiation and electrical testing, these dimensions were re-measured in the SEM to obtain accurate *I*_R_ values in case of beam drift throughout the procedure.

## Results and Discussion

As shown in Figure 1c, the localized irradiation (dose = 10^17^ ions cm^−2^, *I*_R_ = 7%) causes a notably higher electrical conduction to emerge in the monolayer MoS_2_ device. The output current (*I*_ds_) increases approx. fivefold for the same drain–source bias (*V*_ds_) when compared with the non-irradiated device. After the helium ion beam exposure, the transfer characteristics, Figure 1d, reveal a clearly reduced electrostatic response to the applied gate field. The FET channel cannot be effectively turned off in the tested bias range, with significant drain currents persisting even at *V*_g_ = −60 V. This leads to a sharp increase in the subthreshold swing and a large shift of *V*_th_ towards negative gate bias values (Δ*>* 10 V), thus deepening the depletion-mode n-type channel functionality. This observed near-degenerate doping behavior is typical of an increased concentration of sulfur vacancies in defect-rich MoS_2_ [9,20–21]. We note here, that a recent high-energy irradiation study has also called for attention to charge traps generated in the underlying oxide, as the source of donor states in the FET channel [35]. Further studies on flakes decoupled from the substrate need to be performed to clarify the exact origin of the threshold voltage shift in TMDs irradiated at moderate beam energies.

**Figure 2:**
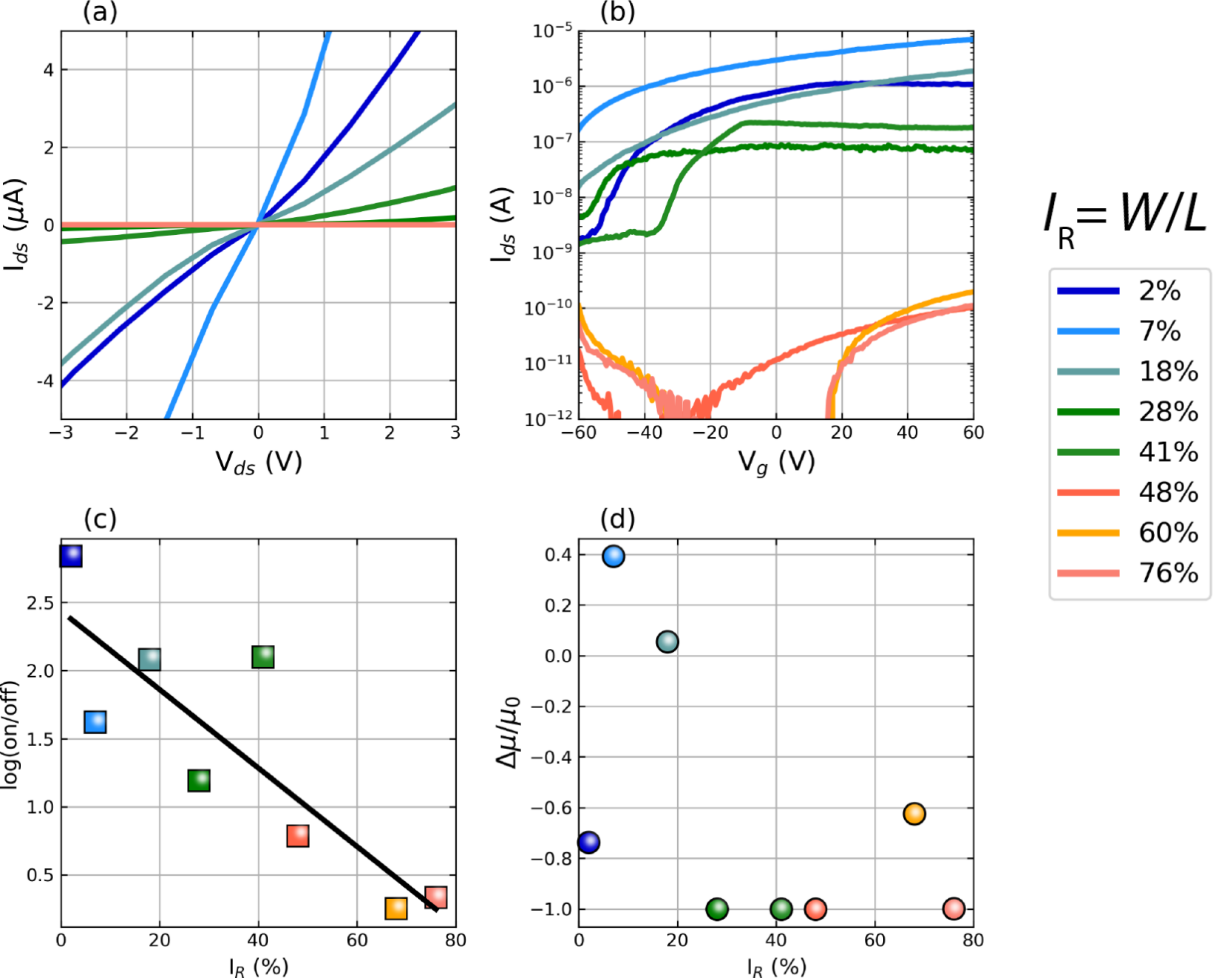
Effects of changing the irradiated area on the performance of monolayer MoS_2_ FETs. Note that all the plots share the same color legend on the right. (a) *I*–*V* and (b) gate sweeps of different devices with varying *I*_R_. (c) Semi-log plot of the extracted electron branch on/off ratios corresponding to each gate curve in (b). The black line is a linear fit to the semi-log data. (d) Changes in the field-effect mobility, μ, relative to as-made device mobilities, μ_0_, extracted from transfer curves in (b).

The effects of increasing *I*_R_ are evident from changes to the recorded output and transfer characteristics in Figure 2a,b. The data are organized into three groups, marked blue, green, and red, corresponding to small (2–18%), medium (28–41%) and large (48–76%) *I*_R_ values, respectively. The *I*_ds_–*V*_ds_ curves, taken at *V*_g_ = 0 V, demonstrate an increase in the channel conductance between the *I*_R_ = 2% and *I*_R_ = 7% irradiations, followed by a continuing drop when *I*_R_ *>* 18%. For small values of *I*_R_ (blue curves in Figure 2b), the FET experiences a large *V*_th_ shift (Δ *>* 10 V) to negative gate biases, a lowered on/off ratio (approx. 10^2^), and an increased conductance relative to the untreated device. As *I*_R_ is increased into the green (28–41%) and red (48–76%) groups, the device conductance drops heavily, accompanied by positive-bias *V*_th_ shifts, while the on/off ratio is also seen to decrease roughly exponentially. This is demonstrated in Figure 2c, which tracks the log-transformed on/off ratio as a function of *I*_R_ with a good linear correlation (*R* = −0.85) from the semi-logarithmic fit. This suggests that an optimal ion irradiation strategy for improving the channel conductance, i.e., when the areal channel exposure is close to 10%, needs to be a balance between introducing significant n-dopant densities and suppressing the unwanted crystal structure amorphization which results from high irradiation doses. At high *I*_R_ values (red group), we observe the emergence of a weak ambipolar response in our transfer curves. At these ratios, the device starts to enter a regime where more than half of the channel has been treated with the ion beam. Thus, we expect a dominant contribution of oxygen-containing atmospheric adsorbates (known p-type dopants in MoS_2_) in saturating the vacancy sites created by the ion beam, allowing for residual hole conduction in the newly formed effective medium channel [36–38]. However, the role of other impurities such as hydrogen and carbon from adventitious surface hydrocarbons in the observed p-doping ought to also be considered in future studies. For a given delivered dose, the ion beam provides a high concentration of effective adsorption sites for atmospheric p-dopants (or adventitious hydrocarbons) within the area defined by the spread of the Gaussian probe extension. The probe has spatially trailing lower-dose tails, the damage of which extends to more than 10 nm and may induce additional n-doping with no complementary adsorbant saturation, i.e., by creating unsaturated SVs in the bottom sulfuric layer [34,39]. A larger irradiation area will thus provide more effective p-doping sites while stifling the n-doping response as the probe tails begin to extend past the FET channel. In addition, low-dose trail areas will be irradiated repeatedly by adjacent line scans, increasing the effective delivered dose and shifting that region to a more acceptor-rich area. This leads to the observed ambipolar gate response for large *I*_R_ values. The formation of these back-to-back heterojunctions may result in unconventional charge transport phenomena if the dose of the irradiation is varied across several orders of magnitude [9], which we leave for future studies.

Figure 2d charts the effect of *I*_R_ on the change in the carrier field-effect mobility, μ, of the irradiated device relative to its as-fabricated mobility, μ_0_. Extracted from the linear region of the transfer curve, μ is seen to improve by up to 40% in two out of three devices in the blue region. For the green and red regions of *I*_R_, μ is always seen to worsen as the area of the irradiated channel is increased. μ is expected to drop heavily as the rate of electron scattering rises with the increased defective channel area. The improved mobility at *I*_R_ ≈ 10% indicates the screening of scattering centers by the increased carrier concentrations induced by SV doping, which leads to the increased conductance observed in Figure 2a. We note that our measurements were performed in a two-probe geometry. Thus, the absolute values of μ extracted here (approx. 1 cm^2^ V^−1^ s^−1^) are limited by the contact resistance between the gold and the MoS_2_ [40].

**Figure 3:**
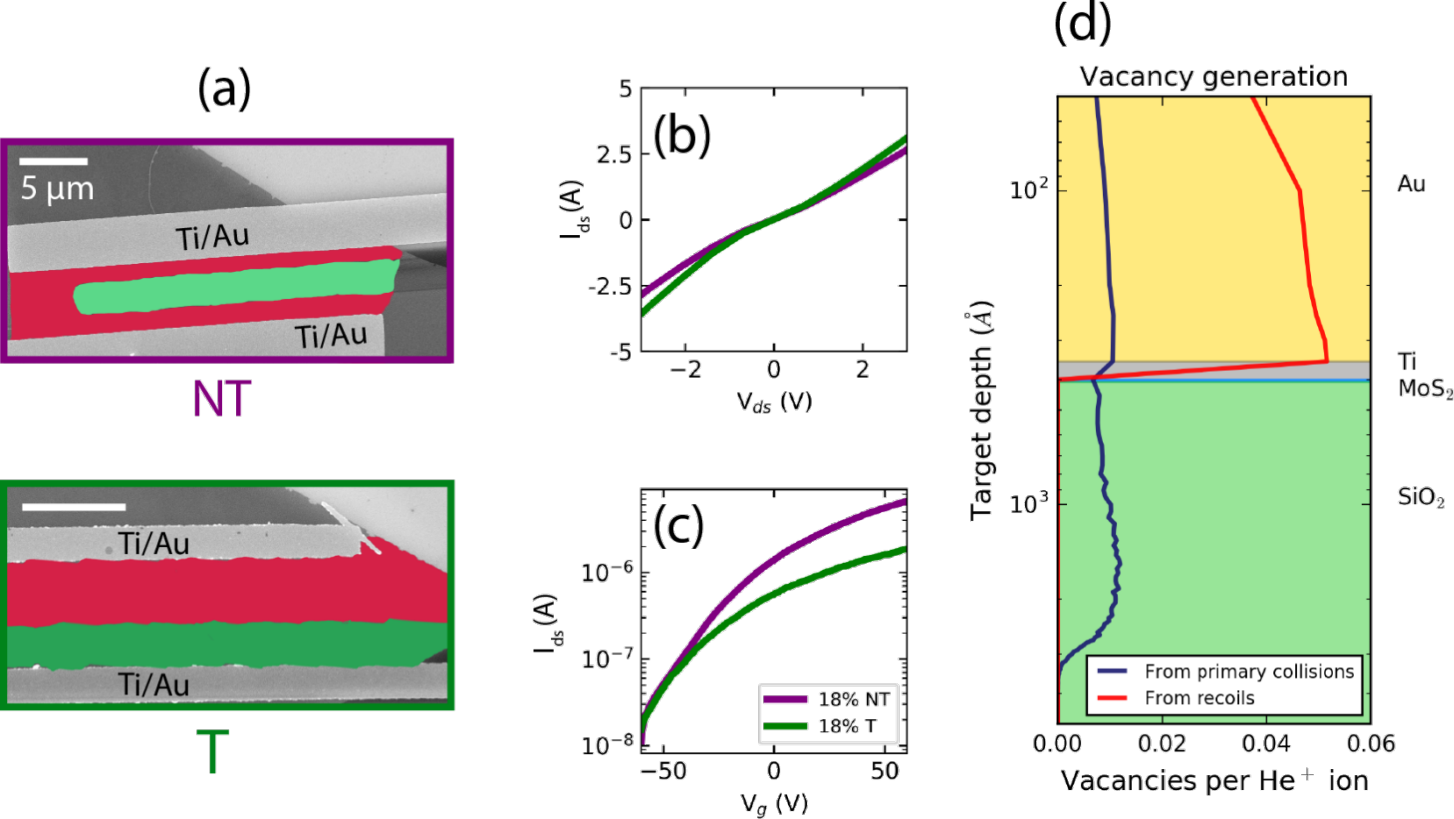
(a) Example SEM images of electrode non-touching (NT) and touching (T) devices with a similar *I*_R_. The colored green area is the channel region damaged by the He^+^ ion beam, while the red area is the non-irradiated MoS_2_ channel. Scale bars are both 5 μm. (b) Output and (c) transfer curves of devices with *I*_R_ = 18% from the NT and T categories, highlighting the deleterious effect of electrode irradiation on the current injection. (d) Simulation of the S vacancy yield generated as the He^+^ ion beam penetrates the device at the contact stack, indicating damage to the metal–semiconductor interface.

We now consider the effect of irradiating the metal–semiconductor interface. We treated two FETs within each *I*_R_ regime. For one of the devices in each pair a single electrode interface was also irradiated. SEM images in Figure 3a show the irradiated channel areas colored in green and the non-irradiated MoS_2_ channel areas in red, on example devices in the high *I*_R_ regime. In all three *I*_R_ regimes, allowing one of the electrode–MoS_2_ interfaces to be damaged by the helium ion beam leads to a drop in the device conductance, as evident in Figure 3b for the representative case of medium *I*_R_ values. Moreover, the conductance of the device where the electrode–MoS_2_ interface was treated with the ion beam is lower than the conductance of the as-fabricated non-irradiated device. The transfer curves in Figure 3c indicate that as the device approaches the strong inversion regime, the electrode–channel interface damage (green curve) inhibits high drain currents in the FET, in contrast to the case of no electrode damage (purple curve).

We simulated the atomic vacancy yield per each delivered ion as a function of target penetration depth on the 35 nm-Au/5 nm-Ti/0.7 nm-MoS_2_/285 nm-SiO_2_ stack [41]. As evident from Figure 3d, the sulfur sputtering yield at the Ti–MoS_2_ interface is very close to that of unencapsulated MoS_2_ [15], indicating notable damage to the interface at this delivered dose. It may be expected that an increase in the Schottky barrier height will occur if the normally pinned Fermi level [42] is now a function of the physical state of the beam-altered metal–semiconductor interface. Ion beam pre-treatment of the contact region before metal deposition increases the concentration of dangling bonds available for molecular hybridization when the contact metal is deposited [32]. As we are treating an already hybridized interface, we suspect that the formation of point defects therein, such as migrated interstitials and antisite defects, will serve to trap carriers at the interface and will reduce the crowded injection current at the contact [43]. This may be empirically confirmed with a combination of low-temperature electrical characterization and capacitance measurements [44–45] in future work.

## Conclusion

In summary, we have studied the effects of varying the irradiated channel area of helium ion-treated monolayer MoS_2_ FETs. Introducing a small number of defects into the material (approx. 10% of irradiated-to-pristine channel area) can serve to improve the charge carrier mobility and the electrical conductance. We found that irradiating the electrode–MoS_2_ interface was deleterious to the performance of the FET, with a conductance drop noticed for each of the areal irradiation regimes. Our work demonstrates that by tuning the helium ion irradiation strategy, and localizing the exposure to specific sites, the electronic characteristics of on-dielectric MoS_2_ FETs can be well-controlled in the monolayer limit. Post-metallization irradiations need to be finely controlled to ensure that the hybridized metal–semiconductor interface is not disturbed, otherwise the drive current in ion beam-treated 2D FETs will be limited for certain applications.
